# Lingual fasciculation: A point of call for the diagnosis of amyotrophic lateral sclerosis

**DOI:** 10.1002/ccr3.7560

**Published:** 2023-06-17

**Authors:** L. Galeazzi, J. Holzman, M. Mondoloni, J. Rochefort

**Affiliations:** ^1^ Department of Oral and Dental Medicine, Pitié‐Salpêtrière Hospital Université Paris Cité, UFR Odontologie Paris France

**Keywords:** ALS, amyotrophic lateral sclerosis, EMG, lingual fasciculations, MUS

## Abstract

A 60‐year‐old female patient, with no notable medical history, was referred by the internal medicine department for a dry mouth workup. The clinical examination revealed an absence of dryness, and the presence of lingual fasciculations, associated with difficulties in mastication and phonation. These symptoms appeared spontaneously 9 months before the consultation, after leaving confinement. Given the presence of lingual fasciculations, the diagnostic hypothesis of a neurological pathology, in particular amyotrophic lateral sclerosis (ALS), was suspected. After performing an electromyogram (EMG), the diagnosis of ALS was retained. Riluzole treatment was then started, and physical therapy sessions were scheduled. Riluzole allows an average gain of 4 to 6 months of life expectancy. Speech therapy and physical therapy allow to maintain the functions as long as possible and to improve the end‐of‐life conditions. The interest of early detection of ALS allows delaying the progression of the disease.

## Graphical abstract

Photographs showing ALS symptoms: fasciculations, lingual mobility limitations, perlèches.

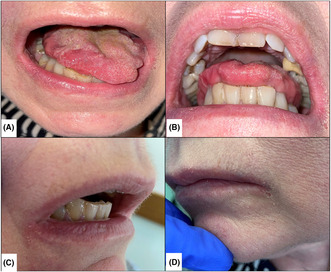

## 1 INTRODUCTION

Amyotrophic lateral sclerosis (ALS) or Charcot's disease is the most common pathology affecting motor neurons. It is characterized by a progressive degeneration of motor neurons in the brain and spinal cord. People with ALS progressively loses connection between muscles and brain. They lose progressively their ability to walk, talk, eat, and eventually breathe. Unfortunately, no curative treatment exists for this disease and death occurs in 8 out of 10 cases in the context of respiratory failure.^1^ Its incidence in France is 2.7 new cases per 100,000 inhabitants (peak incidence between 50 and 75 years of age), and in the world, in 2020, more than 5000 people were diagnosed by year. The average survival is 3 to 4 years after the onset of symptoms. In 10% of cases, the form is familial, and the remaining 90% are sporadic. In both cases, there are two types of development of the pathology: the bulbar and the spinal form.^2^

One of the early clinical signs of this pathology is the presence of fasciculation, especially in the tongue, which can be diagnosed by dentists. A lingual fasciculation is characterized by involuntary, uncontrolled, and constant movements of the tongue. They sometimes occur suddenly, and cause sensations of dry mouth, difficulty in phonation, and dysphagia which leads patients to consult their dentist or their doctor. Then, dentists can be in the front line for the detection, diagnosis, and orientation of ALS patients.

## 2 CASE PRESENTATION

A 60‐year‐old female patient consulted the oral surgery department of the Pitié‐Salpêtrière Hospital, referred by the internal medicine department for an assessment of oral dryness. The patient reported that for the past 9 months, since the end of lockdown due to COVID‐19, she had a sudden sensation of dry mouth, leading to changes in language and discomfort when chewing. She also reported a sensation of dry eyes. The patient had a history of hypertension (treated with Bipreterax 10 mg 1 cp/d) and hormone replacement therapy. In addition, she did not consume alcohol or tobacco and had no allergies.

It was suspected a dry syndrome or a Gougerot–Sjögren's syndrome (GSS) (a systemic autoimmune disease characterized by involvement of the exocrine glands, particularly the lacrimal and salivary glands, characterized by xerostomia). So, an accessory salivary gland biopsy (ASGB) was performed, which consists of the removal of accessory salivary glands from the lip and its analysis in the anatomopathological department. The result was negative for GSS.

**FIGURE 1:**
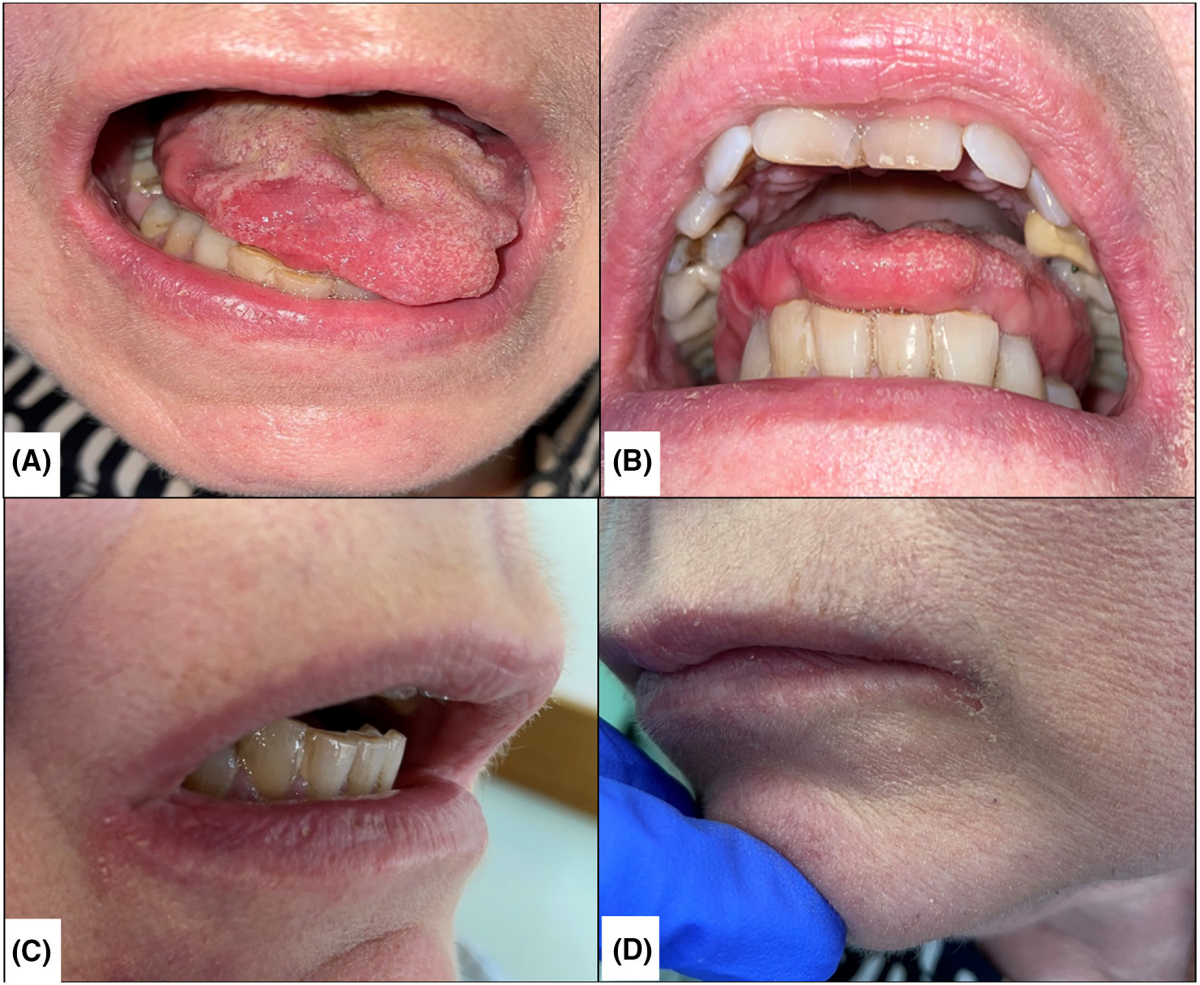
Photographs of the patient's clinical examination. (A): Lingual deviation and limitation of protrusion; (B): Limitation of mouth opening; (C and D): Bilateral perlèches due to salivary incontinence (swallowing defect).

On clinical examination, we found a non‐dry mouth, with normal salivary flow in the 4 ostia. However, we were able to objectify a frank speech impairment, an atrophic tongue, presenting lingual fasciculations (visible and involuntary micromovements of the tongue; see Video S1), associated with depapillated areas on the right and left sides of the back of the tongue. We also noted a defect of lingual protraction (Figure 1A), and of lingual mobility, the patient not being able to touch her palate nor her cheeks, associated with a limitation of the mouth opening (Figure 1B) and bilateral perlèches in connection with a salivary stagnation due to a defect of swallowing (Figure 1C,D). All of this clinicals observations explain the difficulty for the patient to talk and eat normally. These symptoms show a probable damage of the motor neurons.

The whole of this clinical examination, in particularity the presence of lingual fasciculations and the absence of other family neuronal pathologies, allowed us to pose the diagnostic hypothesis of an amyotrophic lateral sclerosis.

Following this consultation, the patient was urgently referred to the neurology department where a workup was performed, with the realization of an electromyography which highlighted arguments for a diffuse peripheral motor neuron damage. A cerebral MRI was also performed to eliminate another cause such as a stroke or an internal hemorrhage. A genetic search (for the C90RF72 and SoD1 genes) was also performed.

All these examinations confirmed a bulbar form of ALS.

From then on, physiotherapy and speech therapy sessions were scheduled in order to maintain motor functions as long as possible. Since the diagnosis made 18 months ago, the global impairment has increased and extended to the lower limbs. At present, the patient can no longer speak and has a loss of autonomy in her daily life. She required a gastric tube because of her difficulties in feeding herself. She lost 10 kg in weight in 1 year. Today, the patient is severely handicapped from a motor point of view but retains her cognitive functions.

## 3 DISCUSSION

The detection of the patient's lingual fasciculations allowed the diagnosis of ALS, as this symptom is characteristic of the bulbar onset form.

To confirm this diagnosis, electromyography (EMG) is the standard method. However, due to its invasiveness, EMG is difficult and painful for the lingual muscles, making it challenging to examine a wide range of muscles simultaneously and unsuitable for short‐term repetition. In contrast, muscle ultrasonography (MUS) has proven to be an excellent alternative for characterizing fasciculations.^3^ For a long time, the method of detection of fasciculations has been electromyography (gold standard); however, this method is long and poorly tolerated by the patients; ultrasonography could eventually replace it, as it is faster and more comfortable. According to Noto & al., the optimal scanning time is 60s for a 40*40 mm area.^4^

Also, genetic tests allow to study the possibility of transmission of the disease, the goal being to establish a predictive factor for diagnosis on the descendants. It is hoped that research in ALS will lead to a potential therapeutic approach, perhaps through the use of gene therapy.

Lingual fasciculations are not only caused by ALS, but other etiologies are also possible such as Machado–Joseph disease, Brown Vialetto Van Laere syndrome (or spinocerebellar ataxia type 3), chronic inflammatory demyelinating polyneuropathy, bulbospinal amyotrophy (or Kennedy's disease), familial amyloid transthyretin neuropathy, osmotic demyelination syndrome, or organophosphate poisoning. Since the presence of lingual fasciculations is mostly due to amyotrophic lateral sclerosis, it is rare to have to consider other diagnoses. However, this can sometimes be misleading, as has been reported in some clinical case reports or studies.^5^, ^6^, ^7^, ^8^, ^9^, ^10^, ^11^

This case report illustrates the importance of a complete examination of the oral cavity. This could be done by every doctor (physician, oral surgeon, dentist). Indeed, since this patient had been complaining of dry mouth for 8 months, the practitioner in charge of the case, who was not a specialist of the oral cavity, set up research and investigations related to syndromes that could cause this type of symptoms, such as Gougerot–Sjögren syndrome, for which reason a biopsy of the accessory salivary glands had been performed. In addition, the onset of symptoms was probably less marked, which led to diagnoses being erroneous and delayed for several months. It is therefore essential to systematically perform a complete examination of the oral cavity. This highlights the importance of a multidisciplinary management in cases of multiple clinical manifestations. If the setting up of multidisciplinary management networks is more and more favored, in particular by the multidisciplinary consultation meetings (RCP), it does not always leave room for the oral cavity specialists which, unfortunately, as in the case of our patient, leads to diagnostic orientation errors.

In addition, it is important to ask about family history when interviewing the patient, as 10% of ALS cases are familial in origin.^2^

Finally, there is an ALS functional assessment scale (ALSFRS‐R), which has become a clinical prognostic marker for clinical and research purposes in amyotrophic lateral sclerosis (ALS). Some teams are trying to develop tools to predict disease progression; however, because some patients have a slow rate of progression, attempts at models are not yet applicable. This may be attributed to the genetic heterogeneity of ALS; a genetic factor should be added to the equation.^12^

Once diagnosis of ALS established, the administration of Riluzole can be initiated. Riluzole has become the primary treatment for ALS due to its ability to inhibit the release of glutamate, which is believed to cause neuronal damage by reaching toxic levels within the body.^13^ Through a review of the literature, it has been established that Riluzole can be considered a disease‐modifying drug for ALS. It increases life expectancy by 4 to 6 months on average.^14^ It is not a real curative treatment; it just delays the progression of ALS.

However, the bulbar phenotype of ALS has the worst prognosis, owing to which patients are unable to survive for a period greater than 2 years.^15^

The limit of this case report is that the oral surgery department had specialist of Oral dermatology, who were trained on this disease and his symptoms, but few people are trained to detect lingual fasciculations, and less for ALS. This case illustrates the few realizations of a systematic oral examination by all physicians. For this patient, physicians of the department of Internal Medicine did not really examine the oral cavity. Also, the lack of knowledge of the first signs of ALS, such as lingual fasciculations and the genes caused by them, is one of the limitations in the diagnosis of ALS.

## 4 CONCLUSION

Early diagnosis of ALS is important to optimize patient management. As one of the first clinical signs is the presence of lingual fasciculations, the dental surgeon (and moreover all physicians) may be led to detect ALS and should be trained to detect them. The diagnosis can then be confirmed by ultrasonography, which is better supported than electromyography. Once the diagnosis is confirmed, accompanying therapies must be started as soon as possible.

We can say that it is essential to systematically perform a complete examination of the oral cavity, it could avoid diagnostic orientation error. This case highlights the importance of a multidisciplinary management in cases of multiple clinical manifestations.

## AUTHOR CONTRIBUTIONS

**L. Galeazzi:** Writing – original draft. **J. Holzman:** Writing – review and editing. **M. Mondoloni:** Writing – review and editing. **J. Rochefort:** Resources; writing – review and editing.

## CONFLICT OF INTEREST STATEMENT

All authors declare that they have no conflicts of interest.

## CONSENT

Written informed consent was obtained from the patient to publish this report in accordance with the journal's patient consent policy.

## Supporting information


Video S1:

## Data Availability

Data sharing not applicable to this article as no datasets were generated or analysed during the current study
